# Benefits of Reducing Prenatal Exposure to Coal-Burning Pollutants to Children’s Neurodevelopment in China

**DOI:** 10.1289/ehp.11480

**Published:** 2008-07-14

**Authors:** Frederica Perera, Tin-yu Li, Zhi-jun Zhou, Tao Yuan, Yu-hui Chen, Lirong Qu, Virginia A. Rauh, Yiguan Zhang, Deliang Tang

**Affiliations:** 1 Department of Environmental Health Sciences, Columbia University, New York, New York, USA; 2 Columbia Center for Children’s Environmental Health, Columbia University, New York, New York, USA; 3 Chongqing Children’s Hospital, Chongqing University of Medical Sciences, Chongqing, China; 4 School of Public Health, Fudan University, Shanghai, China; 5 School of Environmental Science and Engineering, Shanghai Jiao Tong University, Shanghai, China

**Keywords:** China, coal burning, lead, neurobehavioral development, PAH-DNA adducts, prenatal

## Abstract

**Background:**

Coal burning provides 70% of the energy for China’s industry and power, but releases large quantities of polycyclic aromatic hydrocarbons (PAHs) and other pollutants. PAHs are reproductive and developmental toxicants, mutagens, and carcinogens.

**Objective:**

We evaluated the benefit to neurobehavioral development from the closure of a coal-fired power plant that was the major local source of ambient PAHs.

**Methods:**

The research was conducted in Tongliang, Chongqing, China, where a coal-fired power plant operated seasonally before it was shut down in May 2004. Two identical prospective cohort studies enrolled nonsmoking women and their newborns in 2002 (before shutdown) and 2005 (after shutdown). Prenatal PAH exposure was measured by PAH–DNA adducts (benzo[*a*]pyrene–DNA) in umbilical cord blood. Child development was assessed by the Gesell Developmental Schedules at 2 years of age. Prenatal exposure to other neurotoxicants and potential confounders (including lead, mercury, and environmental tobacco smoke) was measured. We compared the cohorts regarding the association between PAH–DNA adduct levels and neurodevelopmental outcomes.

**Results:**

Significant associations previously seen in 2002 between elevated adducts and decreased motor area developmental quotient (DQ) (*p* = 0.043) and average DQ (*p* = 0.047) were not observed in the 2005 cohort (*p* = 0.546 and *p* = 0.146). However, the direction of the relationship did not change.

**Conclusion:**

The findings indicate that neurobehavioral development in Tongliang children benefited by elimination of PAH exposure from the coal-burning plant, consistent with the significant reduction in PAH–DNA adducts in cord blood of children in the 2005 cohort. The results have implications for children’s environmental health in China and elsewhere.

China’s vast industrial network and power plant system rely on coal for approximately 70–75% of their energy needs (Economy 2003; Zhang et al. 2002). Coal burning in China is the major source of ambient poly-cyclic aromatic hydrocarbons (PAHs). PAHs are also present in tobacco smoke and charred foods. Molecular and epidemiologic studies show that fetuses and infants are more susceptible than adults to environmental toxicants including PAHs (Perera et al. 2005), lead [Agency for Toxic Substances and Disease Registry (ATSDR) 2005], and mercury (ATSDR 1999). Experimentally, benzo[*a*]pyrene (BaP), a representative PAH, is a reproductive toxicant (Archibong et al. 2002), producing neurodevelopmental effects including decreased motor activity, neuro-muscular, physiologic and autonomic abnormalities, and decreased responsiveness to sensory stimuli (Saunders et al. 2002, 2003; Wormley et al. 2004b). In studies in Europe, the United States, and China, prenatal exposure to PAHs has been associated with reduced fetal growth (Choi et al. 2006; Perera et al. 1998, 2003; Š rám et al. 2005; Tang et al. 2006) and developmental deficits (Perera et al. 2006). In addition, PAHs are mutagenic and carcinogenic, including via transplacental exposure (Bostrom et al. 2002; Bulay and Wattenberg 1971).

PAH–DNA adducts reflect individual variation in exposure, absorption, metabolic activation, and DNA repair; they therefore provide an informative biologic dosimeter that has been associated with risk of cancer and developmental impairment (Bartsch et al. 1983; Perera et al. 2007; Tang et al. 2001). Here we use adducts as a measure of exposure rather than as a mechanistic marker. PAH–DNA adduct concentrations in cord blood have been shown to increase across a gradient of ambient PAH exposure, albeit with substantial interindividual variation (Perera et al. 2005; Whyatt et al. 1998). Cord blood adducts in the 2002 Tongliang cohort (mean, 0.32 per 10^8^) were significantly higher than those in cohorts of newborns in the United States and Poland (Perera et al. 2005). As previously reported, in the 2002 Tongliang, Chongqing, China, cohort, PAH–DNA adducts in cord blood were associated with reduction of birth head circumference (Tang et al. 2006) and reduced developmental quotients (DQs) at 2 years of age (Tang et al. 2008). As in the present sample, comparison of all cord bloods showed a significant decrease in adduct levels in 2005 (unpublished data).

Lead and mercury are also released by coal burning (Guo et al. 2002; Wang et al. 2006; Zhang et al. 2003) as well as other sources. Both metals are developmental neurotoxicants even at low levels (ATSDR 1999, 2005; Canfield et al. 2003; Needleman et al. 1996) and are potential confounders of associations between PAH–DNA adducts and developmental outcomes.

We tested the hypothesis that comparison of the two cohorts of newborns in Tongliang both followed through 2 years of age would show that elimination of prenatal exposure to coal-burning emissions from the power plant resulted in improved developmental outcomes and in failure to observe the significant effects of PAH–DNA adducts on 2-year development seen in the first cohort, consistent with decreased levels of PAH–DNA adducts in cord blood of children in the second cohort.

## Methods

### Study design

Two identical prospective cohort studies were carried out (pre-and postplant shutdown, respectively). Nonsmoking mothers and their newborns were enrolled at delivery as described (Tang et al. 2008). The newborns were followed through their second birthdays, at which time their intellectual and behavioral development was assessed using the Gesell Developmental Schedules (GDS). Levels of PAH–DNA adducts (specifically BaP–DNA adducts) were measured in umbilical cord blood. Exposure to known neurotoxicants [lead, mercury and environmental tobacco smoke (ETS)] and other potential confounders was assessed by biomarkers or questionnaire.

### Setting

Tongliang, a county in Chongqing Municipality, has a population of around 810,000 and is situated in a basin approximately 3 km in diameter. Before 31 May 2004, a coal-fired power plant located just south of the town center operated every year during the dry season from 1 December to 31 May to compensate for insufficient hydroelectric power during that period (Chow et al. 2006; Tang et al. 2006). The plant was not equipped with modern pollution reduction technology and combusted about 25,000 tons of coal during each 6-month period of operation. In 2002, nearly all domestic heating and cooking units had been converted to natural gas, and motor vehicles were limited in number. Air monitoring analysis carried out as part of the study showed that PAHs of medium molecular weight (168–266) increased by up to 3.5 times during the operational period of the Tongliang power plant (Chow et al. 2006). After the government-mandated shutdown of the plant in May 2004, mean ambient levels of the same PAHs declined significantly (for BaP, *p* = 0.01), as did adducts in cord blood (unpublished data).

### Participants

Subjects were children born to women who gave birth at any one of three major Tongliang County Hospitals located in the Town of Bachuan (informally known as Tongliang City) (representing about 95% of deliveries in Tongliang): the first cohort between 4 March 2002 and 19 June 2002 and the second between 2 March 2005 and 23 May 2005. The women were selected using a screening questionnaire when they checked in for delivery. All women who met the criteria for eligibility (nonsmoker, ≥ 20 years of age, and residence within 2 km of the site of the Tongliang power plant) were invited to participate (the population residing within this radius is about 86,325). Consenting subjects signed the consent form approved by the Columbia University Institutional Review Board and Chongqing University of Medical Sciences. They were administered a questionnaire and contributed a cord blood sample at the time of delivery. Follow-up was conducted by trained research workers who interviewed the mothers about the environmental and health history of their children at 18 and 24 months when the children were brought to the study clinic at 18 and 24 months of age for clinical assessments of health and neurodevelopmental status.

Because physicians first presented the study to the women, all eligible women agreed to participate in the 2002 cohort and only one declined in the 2005 study. The study population is therefore representative of nonsmoking women residing within a 2-km radius of the power plant and delivering in Tongliang. In the 2002 cohort, with a sample size of 150 we observed a 16-point deficit in motor DQ (*p* = 0.043) and a 15-point deficit in average DQ (*p* = 0.047) associated with a log-unit increase in PAH exposure. We therefore expected that a sample size of 150 would allow us to see a similar effect in the 2005 cohort if one existed.

### Personal interviews

A 45-min questionnaire was administered by a trained interviewer after delivery, as described previously (Tang et al. 2006). The questionnaire included sociodemographic information, lifetime residential history, maternal history of active and passive smoking, and exposure during pregnancy to home and workplace chemicals, medications, alcohol, and dietary PAHs. The 6-month interview of the mothers elicited information on children’s health and environment including ETS exposure.

### Biologic sample collection and analysis

As described previously, at delivery umbilical cord blood was collected for PAH–DNA adducts in heparinized Vacutainer tubes and for mercury and lead measurements in EDTA Vacutainer tubes (Tang et al. 2008). Samples were transported to the field laboratory at the Tongliang County Hospital immediately after collection. The buffy coat, packed red blood cells, and plasma were separated and stored at −70°C. Samples were coded and assays were performed on all samples of adequate quantity and quality for analysis.

The laboratory methods have been described in detail (Tang et al. 2008). Briefly, as a validated proxy for PAH–DNA adducts, BaP–DNA adducts in extracted white-blood-cell DNA were analyzed using high-performance liquid chromatography/fluorescence method for BaP tetraols (Alexandrov et al. 1992; Rojas et al. 1994), modified as described (Perera et al. 2007). Samples of whole blood were analyzed for lead and mercury also as described (Tang et al. 2008).

### Measures of child neurodevelopment and covariates

The GDS was selected for comparability to other studies in the Chinese population and because it has been adopted by the Chinese Pediatric Association and is widely used for assessing early child development in China and in other countries (Cui et al. 2001; Jin et al. 2007; Ke et al. 2004; World Health Organization 1999, 2004; Zhang and Li 1994; Zhu et al. 2005). Two-year-old children in the cohort were administered the version of the GDS for 0- to 3-year-old children adapted to the Chinese population (Beijing Mental Development Cooperative Group 1985). Each child is assigned a DQ in each of the four areas: motor, adaptive, language, and social. The standardized mean (± SD) of the DQ is 100 ± 15; a score < 85 indicates developmental delay (Hudon et al. 1998). A study by Jin et al. (2007), which also used the GDS, showed means in the same range as those in our study. Testing was conducted by physicians in the same group who were certified in the GDS to maximize reliable assessment and valid interpretation. Therefore, both interexaminer and intraexaminer variability were minimal.

Research workers abstracted relevant information on covariates from maternal and infant medical records after delivery such as date of delivery, gestational age, and sex of newborn. Other covariates were derived from questionnaire data on socioeconomic status and environmental exposures.

### Statistical analysis

The statistical methods have been previously described with respect to analysis of the 2002 cohort (Tang et al. 2008) and are briefly summarized here. Analysis of the 2005 cohort followed the same procedures. The main exposure of interest was PAH–DNA adducts in cord blood. As before (Tang et al. 2008), adducts were treated as a continuous variable, with nondetectable samples assigned a value of 0.125 per 10^8^ (midway between 0 and the detection limit of 0.25). Lead and mercury values were dichotomized at the median to minimize the influence of outliers. In multiple regression analyses, age-adjusted DQs in the motor area, adaptive area, language area, social area, and the average of these four DQs served as the outcome variables. In logistic regression, the outcomes were developmental delays in the respective areas. As in our prior analyses, we included as covariates sex, gestational age, maternal education, ETS (hours of exposure/day), and lead. Mercury and exposure to chemicals during pregnancy were not included, because neither was a contributor to DQ at the level of *p* ≤ 0.1 (Tang et al. 2008). We did not have direct measures of postnatal PAH–DNA adducts or lead but were able to adjust for postnatal ETS exposure.

We first compared the two cohorts with respect to developmental outcomes (mean DQ scores and frequency of developmental delay) by performing unadjusted analyses using *t*-test or Fisher’s exact test as appropriate. Multiple linear regression and logistic regression were used to test whether the DQ means or odds ratios (ORs) for developmental delay differed significantly between the two cohorts after adjustment for relevant covariates (prenatal ETS, mother’s education, gestational age, and sex) and further including cohort as a covariate. The associations between adducts and developmental outcomes were examined by multiple linear regression and logistic regression as described. In additional exploratory analyses, we tested whether the relationships between cord adducts and DQ and between cord adducts and developmental delay were the same for the two cohorts by including an interaction term (cohort × PAH–DNA) in models combining the two data sets.

## Results

Table 1 provides details of the enrollment and retention of participants in both cohorts. The 2-year retention rate for the first cohort was 88.7% (150 mother–child pairs enrolled, 133 retained). The retention rate for the second cohort was 77.2% (158 pairs enrolled, 122 retained). All 2-year-olds who remained in the cohorts at that time point were administered the GDS. One hundred ten subjects in 2002 and 107 subjects in 2005 had complete data required for hypothesis testing. The sociodemographic, clinical, and environmental characteristics of the cohorts are provided and compared in Table 2. The subjects included in the analysis did not differ (*p* < 0.05) from those not included with respect to these characteristics except that in the 2005 cohort subjects included in the model had a higher mercury level. The two cohorts differed with respect to maternal age. As reported in the full cohorts (unpublished data), the mean PAH–DNA adduct level and percent of newborns with detectable adduct levels were significantly reduced in the 2005 cohort (*p* < 0.001). The 2005 mean for PAH–DNA adducts in the present sample was 0.20 per 10^8^, and 51% of samples had detectable adducts. Mean lead and mercury concentrations did not differ between 2002 and 2005.

Table 3 compares the distribution of DQs for both cohorts. As in the 2002 cohort, in 2005 all DQ domains were significantly intercorrelated (*p* < 0.01), with *r*-values ranging from 0.40 to 0.83. Unadjusted comparisons of the mean scores showed that mean DQs, except for language area, were higher in 2005 but not significantly so. After adjustment for relevant covariates, the only significant difference in mean score was the social area DQ (*p* = 0.033) (Table 3). The frequencies of developmental delay in all DQ areas except for language were reduced in 2005 compared with 2002; before adjustment, the difference was significant for delay in the motor area (*p* = 0.033). After adjustment, a significant difference in the frequency of delay in the motor area (*p* = 0.017) remained.

The results of multiple regression analysis are shown in Table 4. In the 2002 cohort, cord adducts showed significant inverse associations with the DQ in the motor area before and after adjusting for cord lead level, ETS, sex, gestational age, and maternal education level [unadjusted β = −13.78; 95% confidence interval (CI), −29.18 to 1.63; *p* = 0.082; adjusted β = −16.01; 95% CI, −31.30 to −0.72; *p* = 0.043]. In 2002, cord adducts were also associated with the average DQ (adjusted β = −14.58; 95% CI, −28.77 to −0.37, *p* = 0.047). In contrast, in the 2005 cohort, cord adducts were not significantly associated with any of the DQs before or after adjusting for the same covariates (for motor area DQ, adjusted β = −5.90; 95% CI, −24.96 to 13.17; *p* = 0.546) and average DQ (β = −12.38; 95% CI, −28.95 to 4.21; *p* = 0.146). However, in 2005, associations for all DQs remained inverse, albeit not statistically significantly. Whereas in the 2002 cohort, by logistic regression analysis, a 0.1-unit increase (0.1 adduct/10^8^ nucleotides) in cord adducts was associated with increased odds of being developmentally delayed in the motor area (OR = 1.91; 95% CI, 1.22 to 2.97, *p* = 0.004), this association was not seen in 2005 (OR = 2.06; CI, 0.62 to 6.84, *p* = 0.240), nor were significant associations seen in 2005 between PAH–DNA adducts and developmental delay in the other DQ areas (Table 5). However, the interaction term (PAH × cohort) was not significant in multiple or logistic regression models, which may be attributable to small sample size.

Further controlling for postnatal ETS, in multivariate regression the associations between adducts and DQ in the motor area and average DQ remained significant in the 2002 cohort: β = −16.89; 95% CI, −31.76 to −2.01, *p* = 0.026 and β = −16.56; 95% CI, −31.21 to −1.92, *p* = 0.027, respectively. Also by logistic regression, the OR for adducts and motor area delay remained significant: OR = 2.18; 95% CI, 1.31 to 3.61; *p* = 0.003. Inclusion of postnatal ETS in the 2005 models did not affect the results.

## Discussion

As hypothesized, comparison of the two cohorts of newborns in Tongliang, China, both followed through 2 years of age, has provided evidence that elimination of prenatal exposure to coal-burning emissions resulted in measurable benefits to children’s development. In contrast to the 2002 cohort, in the 2005 cohort we did not observe a significant effect of PAH–DNA adducts on 2-year developmental scores. Consistent with prior analyses of adducts among all newborns (unpublished data), in the present subset the average adduct concentration and the frequency of detectable adducts in cord blood were reduced in the 2005 cohort by 38% and 36%, respectively. The mean cord adduct level in the 2002 cohort (0.32 adducts/10^8^ nucleotides) was significantly higher than in New York City (0.21 adducts/10^8^ nucleotides) or Krakow, Poland (0.28 adducts/10^8^ nucleotides), consistent with the higher ambient PAH exposure in Tongliang (Perera et al. 2005). After the power plant shutdown, the adduct levels in Tongliang in 2005 (0.20/10^8^ nucleotides) were similar to those in New York City (0.21/10^8^).

Whereas PAH–DNA adducts in cord blood were significantly associated with DQ decrements in the motor area and in the average DQ among children who were *in utero* during the power plant operation, these significant associations were not seen among children who were *in utero* after the power plant had been shut down. In the 2002 cohort, adducts were associated with an approximate 2-fold increased odds of developmental delay in the motor area; again, that effect was not seen in the 2005 cohort. However, the observation in the 2005 cohort of inverse, albeit non–statistically significant, associations between adducts and all DQs except for the average DQ suggests that even greater benefits will accrue in the future. PAHs are lipid-soluble compounds (Nickerson 2006), and it is reasonable to expect that the concentrations of PAHs stored in mothers’ adipose tissue and transferrable to the fetus will be reduced over time. In 2007, we enrolled a third cohort of mothers and newborns, which we will follow to determine longer-term benefits of closing the power plant.

The finding of adverse developmental effects in the 2002 cohort is consistent with experimental findings that prenatal exposure to BaP during critical windows of brain development produces a variety of neurodevelopmental effects in the offspring (Wormley et al. 2004a) and with our prior finding in a New York City cohort that prenatal PAH exposure was associated with developmental impairment at age 3 years (Perera et al. 2006). The mechanisms by which PAHs adversely affect child development are not well understood.

Strengths of the study include the prospective cohort designs, the use of a molecular marker of PAH exposure, and the ability to control for potential confounders. The results are internally consistent and generalizable to other nonsmoking Chinese women. A limitation of the study is that we did not have data on postnatal levels of PAH–DNA or metals to permit examination of the impact of postnatal exposure on 2-year cognitive development. Because the power plant was not shut down until May 2004, the subjects in the 2002 cohort continued to receive seasonal exposure to the plant emissions after birth. However, several lines of evidence indicate that fetal development is a period of heightened susceptibility to PAHs and lead (ATSDR 1999, 2005; Perera et al. 2005). We note that adjustment for postnatal ETS exposure did not alter the effect of PAH–DNA adducts. Another limitation of the present study is the small sample size in each cohort, which limited our ability to evaluate interactions between adducts and cohort on development or to assess interactions between pollutants.

Although results are interpretable only on the group level, the improvement in developmental outcomes and the lack of significant associations between PAH–DNA adducts and deficits in development in the 2005 cohort may be educationally meaningful, because compromised function at an early age may have a negative impact on subsequent school performance (Drillien et al. 1988). Within a Chinese population, there was a significant correlation between developmental assessment at 6–12 months on the Gesell and mental development at 6–7 years on the Chinese version of the Wechsler Scales for Children (*p* < 0.01) (Zhou et al. 2004). Continued follow-up of the present cohort will determine whether reduction of prenatal PAH exposure is associated with subsequent measures of cognitive development and school performance.

## Conclusion

In conclusion, these results indicate that an intervention to eliminate emissions from a polluting coal-burning power plant was effective in improving developmental outcomes among children living in Tongliang, Chongqing. Because coal-fired power plants currently produce 75% of China’s electricity and most new plants in China are being built to burn coal, albeit with modern pollution control, the results from the Tongliang study are relevant to the development of other children living in China and have implications for policies concerning energy and public health.

## Figures and Tables

**Table 1:** Enrollment and retention of the cohorts.

	2002	2005
Examined for eligibility (screened)^a^	202	173
Confirmed eligible	150	159
Included in the study^b^	150	158
Completed follow-up^c^	133	122

a In 2002, 52 women were either < 20 years of age, smoked during pregnancy, or resided > 2 km from the site of the power plant. The number of ineligible women (same criteria) in 2005 was 13.

b In 2005, one woman declined to be interviewed.

c Loss to follow-up before child’s age 2 was attributed to work-related moves out of the county (17 women in 2002, 27 in 2005).

**Table 2:** Demographic and exposure characteristics of the cohorts.

Characteristic	2002 (*n* = 110)^a^ Mean ± SD (range) or %	2005 (*n* = 107) Mean ± SD (range) or %
Maternal age (years)*	25.18 ± 3.15 (20.34–34.28)	27.91 ± 4.59 (20.45–37.80)
Maternal education (%)		
< High school	43.6	55.1
≥ High school	56.4	44.9
Sex of newborn (% female)	50.9	44.9
Gestational age (days)	277.35 ± 11.27 (224–294)	276.69 ± 9.19 (250–300)
Cord lead (mg/dL)	3.60 ± 1.59 (0.82–12.93)	3.74 ± 1.50 (1.49–10.82)
Cord mercury (ppb)	6.97 ± 4.43 (2.28–39.72)	6.61 ± 2.77 (1.72–14.23)
Prenatal ETS exposure (hr/day)	0.29 ± 0.59 (0–5.00)	0.30 ± 0.54 (0–3.00)

a Number of subjects with each type of data varies due to missing data.

* *p* < 0.05; comparisons of continuous variables by Mann–Whitney test, and binary variables by chi-square test.

**Table 3:** Comparison of Gesell scores in the two prospective cohorts.^a^

DQ area	2002 Cohort^b^*n* = 110	2005 Cohort *n* = 107
Motor area		
Mean ± SD (range)	97.53 ± 11.47 (65–135)	97.83 ± 7.82 (74–116)
Normal [*n* (%)]	95 (86.4)	102 (95.3)
Developmental delay [*n* (%)]^c^	15 (13.6)	5 (4.7)
Adaptive area		
Mean ± SD (range)	98.71 ± 14.90 (50–124)	101.18 ± 10.96 (76–129)
Normal [*n* (%)]	96 (87.3)	96 (89.7)
Developmental delay [*n* (%)]	14 (12.7)	11 (10.3)
Language area		
Mean ± SD (range)	102.10 ± 12.83 (56–122)	100.47 ± 9.78 (74–127)
Normal [*n* (%)]	99 (90.0)	96 (89.7)
Developmental delay [*n* (%)]	11 (10.0)	11 (10.3)
Social area		
Mean ± SD (range)^d^	99.40 ± 11.79 (57–121)	101.83 ± 6.81 (76–117)
Normal [*n* (%)]	100 (90.9)	104 (97.2)
Developmental delay [*n* (%)]^e^	10 (9.1)	3 (2.8)
Average		
Mean ± SD (range)	99.42 ± 10.74 (57–120)	100.30 ± 7.16 (76–117)
Normal [*n* (%)]	103 (93.6)	105 (98.1)
Developmental delay [*n* (%)]^e^	7 (6.4)	2 (1.9)

a Unadjusted comparisons of DQs between cohorts by *t*-test, percent delay by Fisher’s exact test; adjusted analyses by regression as described.

b This material appears as originally published in Tang et al. (2008).

c Unadjusted, *p* = 0.033; adjusted, *p* = 0.017.

d Unadjusted, *p* = 0.064; adjusted, *p* = 0.033.

e Unadjusted NS (not significant); it is not appropriate to use logistic regression due to small cell count.

**Table 4:** Results of multiple regression analyses of Gesell scores at 2 years of age and PAH–DNA adducts.^a^

DQ area	2002 Cohort β(95% CI), *p*-value	2005 Cohort β(95% CI), *p*-value
Motor area	−16.01 (−31.30 to −0.72) *p* = 0.043	−5.90 (−24.96 to 13.17) *p* = 0.546
Adaptive area	−15.51 (−35.63 to 4.61) *p* = 0.134	−22.06 (−47.70 to 3.59) *p* = 0.095
Language area	−16.64 (−33.73 to 0.46) *p* = 0.059	−20.39 (−42.62 to 1.85) *p* = 0.075
Social area	−9.29 (−25.28 to 6.70) *p* = 0.258	−1.50 (−17.62 to 14.61) *p* = 0.855
Average	−14.58 (−28.77 to −0.37) *p* = 0.047	−12.38 (−28.95 to 4.20) *p* = 0.146

a Model included cord lead level, sex, gestational age, maternal education, lead, and ETS as covariates.

**Table 5:** Results of logistic regression analyses of developmental delay at 2 years of age and PAH–DNA adducts.^a^

DQ area	2002 Cohort OR (95% CI),^b^*p*-value	2005 Cohort OR (95% CI), *p*-value
Motor area	1.91 (1.22 to 2.97) *p* = 0.004	2.06 (0.62 to 6.84) *p* = 0.240
Adaptive area	1.16 (0.76 to 1.76) *p* = 0.500	1.78 (0.79 to 4.00) *p* = 0.161
Language area	1.31 (0.84 to 2.05) *p* = 0.234	2.34 (0.96 to 5.71) *p* = 0.061
Social area	1.52 (0.93 to 2.50) *p* = 0.095	3.38 (0.59 to 19.35) *p* = 0.171
Average	1.67 (0.93 to 3.00) *p* = 0.088	NA^c^

NA, not available.

a Model included cord lead level, sex, gestational age, maternal education, and ETS as covariates.

b The ORs for cord adducts presented in this table represent the effect of a 1-unit (0.1 adduct/10^8^ nucleotides) increment in cord adducts.

c SPSS statistical software (SPSS Inc, Chicago, IL, USA) failed to provide accurate estimates.
